# Prevention of Cholera

**Published:** 1889-12-28

**Authors:** 


### PREVENTION OF CHOLERA.

The *Sei-i-Kwai Medical Journal* is the title of a professional paper now published monthly in Japan. Sei-i-Kwai means, we believe, the society for the advancement of medical science. Among the articles in a recent number of this journal is a report on preventive measures against cholera by Surgeon-Major Ishiquro Tadanoïi, of the Japanese Army. The surgeon-major states that cholera is not native to the country, but was always brought from China, India, or other places. The preventive measures suggested appears to be based on recommendations made by Dr. Koch to the author, and consist principally of inspection, quarantine, and disinfection. What we desire to observe with reference to this article, which doubtless reflects the ideas prevalent on the subject in Japan, is that there is great doubt if we are justified in regarding either India or China as the home of cholera, as the place where it always originates and from whence it always spreads. There was a creed, principally promulgated by the late Surgeon-Major Bryden, when secretary to the Sanitary Commissioner in India, that cholera always originated in the delta of Bengal and spread thence westwards. Elaborate essays were written to prove this. But there was always one serious objection—viz., that investigations as to the supposed onward westward march of cholera were perforce confined to the countries west of India. For little was known of the countries east of India, and no accurate information could be obtained. If this had been possible, it is quite likely that equal reason might have been advanced for an eastern spread of the malady. Although (notwithstanding Koch's comma bacillus) we have not yet discovered the cause of cholera, there is no doubt that it is closely allied to, if not identical with, the malady which we term summer diarrhœa in adults, and *cholera infantum* in children. So many cases of cholera commence as diarrhœa, and so many cases of diarrhœa always occur during a cholera epidemic, that the same agency must be credited as the cause. Cholera of a mild nature occurs in all countries, excepting perhaps in the Arctic regions, and it is not necessary to trace its origin to the maligned delta of Bengal.

